# 

**Published:** 1900-11-10

**Authors:** 


### NOTICE TO CORRESPONDENTS.

All MSS., Letters, Books for Review, and other matters intended for the Editor should be addressed The Editor, "The Hospital," The Hospital Building, 28 & 29 Southampton Street, Strand, W.C.

The Editor cannot undertake to return rejected MS., even when accompanied by stamped directed envelope.

**Notice to Advertisers and Subscribers.**

All Advertisements, Orders for Copies of The Hospital, and Business Communications should be addressed to **THE MANAGER (Not to the Editor),** The Hospital, The Hospital Building, 28 & 29 Southampton Street, Strand, London, W.C.

The Cost of Subscription, post free, is as follows:—Per week, 2½d.; per per quarter, 2s. 8d.; per half-year, 5s. 3d.; per annum, 10s. 6d. Foreign:—Per annum, 15s. 2d., payable, in all cases, in advance.

**NOTICE.—Vol. XXVIII. of "The Hospital" (April to September, 1900), in handsome cloth binding, will shortly be on sale at the Office of this Journal, price, 7s. 6d. Cases for binding the Numbers contained in this Volume 1s. 6d. Index to Vol. XXVIII., 2½d., post free.**

**The Monthly Part for November is now ready. Price 6d., by post 8d.**

### BOOKS RECEIVED.

Edward Arnold.

"Food and the Principles of Dietetics." By Robert Hutchinson, M.D.Ed., M.R.C.P.

T. Fisher Unwin.

"First Aid to the Injured." By Dr. Oscar Bernhard. Translated by Michael G. Foster, M.A., M.D. Cantab.

Isbister and Co.

"The Sunday Magazine," 1900. "Good Words," 1900. Edited by the Very Rev. Donald Macleod, D.D.

### SCRAPS AND GLEANINGS.

The total income of the Bridgwater Infirmary during the past year was £1,596 odd, being £387 less than the expenditure.

The amount of workmen's contributions to the Sunderland Infirmary during the past year totalled £6,287 odd, out of a total income from subscriptions of £8,280.

During the past year the total number of visits paid by the District Nurse of the Doncaster Infirmary was 3,140, a number which is considerably in advance of the visits paid last year.

It is proposed that the new fever hospital for Perth should contain 40 beds, and that they should be apportioned as follows:—30 for scarlet fever, 4 for typhoid, 4 for diphtheria, and 2 for erysipelas.

In order to clear off the deficit of £400 in the funds of the Doncaster Infirmary, it has been decided to open a shilling subscription list through the Press; and also to make a special appeal to the friends of the institution for increased subscriptions.

The Dental Hospital of London, has received a donation of £100 and a new Yearly Subscription of £10 10s. to the "General Maintenance" Fund, and £100 as a special donation towards the amount urgently required for rebuilding the Hospital, from Lord Grimthorpe, Q.C.

Sixteen soldiers from South Africa have been treated during the season at the Alexandra Hospital, Woodhall Spa. Of these, nine have returned to duty cured. The hospital was closed for the season on November 1st, and large additions are to be at once commenced.

The total number of cases treated during the year ending June 30th, 1900, at the Sunderland Infirmary, was 8,612, against 7,754 the previous year. Notwithstanding this increase in the number of patients treated, the number of deaths decreased from 144 last year to 130.

The Building Committee of the Portsmouth Board of Guardians at a recent meeting had before them the question of the cure of consumptive patients by the fresh-air treatment. It was at first proposed to convert two of the existing wards of the infirmary for the purpose, but after some discussion a resolution was unanimously carried to the effect that the Board considered that such accommodation should be provided by sanitary authorities, and that the whole of the Boards of Guardians in the county be requested to combine in petitioning the Hants County Council to provide a sanatorium, to which Boards of Guardians may send patients at a fixed rate.

### The Student of Medicine.

### APPOINTMENTS.

Charles Aymer, M.B.Aberd., C.M., appointed Surgeon and Agent, Calterline, Bervie and Johnshaven. Allan Arthur Bathe, M.A., M.B.Oxon., appointed an Honorary Medical Referee to the Royal National Hospital for Consumption. H. C. Brown, M.R.C.S., L.R.C.P.Lond., appointed Medical Officer for the Tipton West District of the Dudley Union. John Campbell, L.R.C.S., L.R.C.P.Irel., appointed Medical Officer to the Estates of the New London Borneo Tobacco Company, Kudat. W. B. Grannum, L.R.C.P., L.R.C.S.Edin., appointed Medical Officer for the East District of the Luton Union. Charles Herbert Gunson, M.B., Ch.B.Glasg., appointed Resident Surgeon to the North Cambridgeshire Hospital, Wisbech. James Haworth, M.B., Ch.B., appointed House-Surgeon to the Sunderland Infirmary. G. C. Hobbs, M.R.C.S., L.R.C.P., appointed Resident Medical Officer to the Hampstead Hospital. Arthur S. Jaques, L.R.C.P., L.R.C.S.Edin., appointed Medical Officer for the East District of the Gateshead Union. F. C. McKee, B.A., M.B., B.Ch., B.A.O.R.U.I., appointed House-Physician in the Sunderland Infirmary. H. B. Mapleton, M.A.Oxon., M.D.Edin., appointed Medical Officer of Health for the Newton Abbott Rural District. S. H. Merryweather, L.R.C.P., L.R.C.S.Edin., appointed Medical Officer for the Tattershall District of the Horncastle Union. John Moorhead, M.A., M.D., appointed Consulting-Physician to the Royal Hospital, Weymouth. J. H. Naismith, M.B.Glas., appointed District Medical Officer to the Weardale Union. J. E. Platt, M.S.Lond, F.R.C.S., appointed Assistant to the Professor of Surgery in the Owens College, Manchester. Roland A. Stevenson, L.R.C.P.Lond., M.R.C.S.Eng., appointed House-Physician at the Hospital for Consumption, Brompton, S.W. J. B. Thackwell, M.B.Edin., appointed District Medical Officer to the Wandsworth and Clapham Union. John C. Thorowgood, M.D.Lond., appointed Consulting Physician to the Royal National Hospital for Consumption at Ventnor. Robert B. Wild, M.Sc.Vict., M.D.Lond., M.R.C.P., appointed Honorary Physician to the Cancer Pavilion and Home, Manchester. Rivers Willson, Ph.D., L.S.A.Lond., appointed Surgeon to the Oxford Medical Dispensary and Lying-in Charity.

